# Vulnerable Parkin Loss-of-Function *Drosophila* Dopaminergic Neurons Have Advanced Mitochondrial Aging, Mitochondrial Network Loss and Transiently Reduced Autophagosome Recruitment

**DOI:** 10.3389/fncel.2018.00039

**Published:** 2018-02-15

**Authors:** Juliana Cackovic, Susana Gutierrez-Luke, Gerald B. Call, Amber Juba, Stephanie O’Brien, Charles H. Jun, Lori M. Buhlman

**Affiliations:** ^1^Arizona College of Medicine, Midwestern University, Glendale, AZ, United States; ^2^Department of Biomedical Sciences, College of Health Sciences, Midwestern University, Glendale, AZ, United States

**Keywords:** parkin, neurodegeneration, dopaminergic neurons, Parkinson’s disease, autophagy, mitochondria, mitochondrial dynamics, MitoTimer

## Abstract

Selective degeneration of substantia nigra dopaminergic (DA) neurons is a hallmark pathology of familial Parkinson’s disease (PD). While the mechanism of degeneration is elusive, abnormalities in mitochondrial function and turnover are strongly implicated. An Autosomal Recessive-Juvenile Parkinsonism (AR-JP) *Drosophila melanogaster* model exhibits DA neurodegeneration as well as aberrant mitochondrial dynamics and function. Disruptions in mitophagy have been observed in parkin loss-of-function models, and changes in mitochondrial respiration have been reported in patient fibroblasts. Whether loss of parkin causes selective DA neurodegeneration *in vivo* as a result of lost or decreased mitophagy is unknown. This study employs the use of fluorescent constructs expressed in *Drosophila* DA neurons that are functionally homologous to those of the mammalian substantia nigra. We provide evidence that degenerating DA neurons in parkin loss-of-function mutant flies have advanced mitochondrial aging, and that mitochondrial networks are fragmented and contain swollen organelles. We also found that mitophagy initiation is decreased in *park* (*Drosophila parkin/PARK2* ortholog) homozygous mutants, but autophagosome formation is unaffected, and mitochondrial network volumes are decreased. As the fly ages, autophagosome recruitment becomes similar to control, while mitochondria continue to show signs of damage, and climbing deficits persist. Interestingly, aberrant mitochondrial morphology, aging and mitophagy initiation were not observed in DA neurons that do not degenerate. Our results suggest that parkin is important for mitochondrial homeostasis in vulnerable *Drosophila* DA neurons, and that loss of parkin-mediated mitophagy may play a role in degeneration of relevant DA neurons or motor deficits in this model.

## Introduction

A growing body of evidence suggests that parkin, an E3 ubiquitin ligase, plays an important role in mitochondrial integrity. The means by which parkin supports mitochondrial function are less clear. A widely held theory behind the mechanism by which loss of parkin function causes neurodegeneration is that parkin-deficient cells lack the ability, or have impaired ability, to rid themselves of depolarized mitochondria. The increasing presence of damaged mitochondria becomes an insurmountable burden that causes cells to undergo apoptosis (reviewed in Pickrell and Youle, 2015). The highly oxidizing mitochondrial environment warrants efficient mitochondrial turnover to maintain optimal cellular respiration, particularly in cells with high energy demands. Cytosolic parkin can be recruited to depolarized mitochondria in order to initiate mitophagy (Narendra et al., 2008; Hämäläinen et al., 2013). Parkin-mediated ubiquitination of a variety of outer mitochondrial membrane substrates precedes autophagosomal engulfment of the mitochondrion. These ubiquitination events may serve as signals for autophagosome recruitment (Ding et al., 2010; Geisler et al., 2010; Narendra et al., 2010; Chan et al., 2011; Sarraf et al., 2013; Wong and Holzbaur, 2014; Lazarou et al., 2015). Whether the absence of parkin causes DA neurodegeneration as a result of lost or decreased mitophagy *in vivo*, however, is still unknown. Much of the evidence addressing the role of parkin in mitophagy comes from manipulations of overexpression systems in cultured cells, which facilitate elegant live imaging studies. Unlike neurons, immortalized cells rely on glycolysis, rather than oxidative phosphorylation as their main source of ATP (Crabtree, 1929). *Drosophila park* (*Drosophila parkin/PARK2* ortholog) loss-of-function mutants have neuron and muscle degeneration, defective spermatids and dysfunctional mitochondria that have aberrant morphology (Greene et al., 2003; Burman et al., 2012). Interestingly, *park* heterozygous loss-of function flies have motor deficits, but their DA neurons do not degenerate (Chambers et al., 2013). *Park* homozygous mutants that lack the *glutathione S-transferase S1 (GstS1)* gene (encoding antioxidant glutathione s-transferase) have more pronounced phenotypes, and *GstS1* overexpression prevents DA neuron degeneration (Greene et al., 2005; Whitworth et al., 2005). This suggests that increased reactive oxygen species (ROS) load is a major factor in neurodegeneration caused by the absence of parkin. Indeed, advanced aging is regularly associated with increased ROS load (Beckman and Ames, 1998; Jacob et al., 2013). How the absence of parkin yields increased oxidative stress is unclear, but decreased mitophagy and/or proteasome-mediated turnover of mitochondrial proteins may be implicated (Vincow et al., 2013).

We have explored the effects of parkin loss-of-function on mitochondrial network integrity, mitochondrial aging, autophagosome formation and mitophagy initiation in *park* mutant *Drosophila* (*park*^25^) (Greene et al., 2003) protocerebral posterior lateral region 1 (PPL1) and protocerebral posterior medial region 3 (PPM3) DA neurons. These neurons are functionally homologous to those of the mammalian substantia nigra *pars compacta* (reviewed in Strausfeld and Hirth, 2013); however, only PPL1 neurons degenerate in *park* loss-of-function flies (Whitworth et al., 2005). PPL1 neurons can be spared when autophagosome-promoting factor autophagy 8a (atg8a) is exogenously expressed (Burman et al., 2012). In order to address whether autophagosome formation and mitophagy initiation are affected by the *park* mutation, we utilized flies that express autophagosome marker mCherry-atg8a (mCherry-atg8a; Ichimura et al., 2000; Nezis et al., 2009; Shpilka et al., 2011). We generated *park* mutant flies that express a mitochondrially targeted green fluorescent protein (mitoGFP) to measure mitochondrial network integrity (Rizzuto et al., 1995). Mitochondrial aging was observed using *park* mutant flies that express the MitoTimer construct, which encodes a mitochondrially-targeted DsRed variant. MitoTimer excitation and emission spectra shift from green to red as mitochondria age, likely as a result of oxidation at the Tyr-67 residue (Yarbrough et al., 2001; Laker et al., 2014). Increased levels of red MitoTimer also have been used to indicate decreases in mitochondrial turnover (Ferree et al., 2013; Hernandez et al., 2013). Using this *in vivo* model, we provide evidence that degenerating DA neurons may be vulnerable because of selective dependence on parkin-mediated mitophagy.

## Materials and Methods

### *Drosophila* Maintenance and Strains

All flies were maintained on standard cornmeal molasses food at 25°C and 12/12-h light/dark cycle. Flies were transferred to vials containing new food every 3–4 days. The *park^25^* loss-of-function mutants (Greene et al., 2003) were a gift from Leo Pallanck at the University of Washington in Seattle. *W*^1118^–containing control stocks and *Drosophila* harboring *tyrosine hydroxylase (TH)-GAL4* (*P{ple-GAL4.F}*; stock 8848), *upstream activating sequence (UAS)-mitoGFP* (*P{UAS-mito-HA-GFP.AP}3*; stock 8442), *UAS-MitoTimer* (stock 57323), and *UAS-Atg8a* (*P{UASp-mCherry-Atg8a}*; stock 37750) constructs were obtained from the Bloomington *Drosophila* Stock Center at Indiana University, Bloomington (Rizzuto et al., 1995; Friggi-Grelin et al., 2003; Nezis et al., 2009; Laker et al., 2014). All experimental flies were outcrossed to a *w*^1118^ control line. The *park*^25^ mutant genotypes were confirmed via PCR and/or via observation of *park*^25^ loss-of-function phenotype. Flies harboring *UAS-*fluorescent constructs were crossed with those harboring *TH-GAL4* in order to drive expression exclusively into TH-producing cells.

### Confirmation of *park*^25^Alleles Using Polymerase Chain Reaction

Presence and absence of the *park^25^* allele was detected using a standard PCR protocol incorporating primers park25F (5′-GAT TGG CAA CAC TGA AGC-3′) and park25R (5′-CTT TAC CAT CCC CCA ATC AA-3′) designed in the Pallanck laboratory at the University of Washington, Seattle, WA, USA. Wild-type *park* product is 2.3 kb, and the mutant *park*^25^ product is 960 bp. The 2.3 kb wild-type product is not amplified consistently; therefore, park(5) (5′-GAT ACG ACG GGA TTA CAA GGG G-3′) and park(6) (5′-TGT CTT CTA GTA GCA ATG TGA CTT-3′) primers were used to amplify a 272 bp product that is absent from flies harboring the *park*^25^ allele.

### Immunohistochemistry

Fly heads were removed, and brains were dissected on days 5, 10 and 20 post eclosion (PE: the day *Drosophila* emerge from their pupa cases) in phosphate-buffered saline (PBS). Brains were immediately fixed in 3.7% formaldehyde (VWR, West Chester, PA, USA) in PBS for 30 min. Following four washes in 0.3% PBT (PBS containing 0.3% Triton X-100; MP Biomedical, Salon, OH, USA) for 5 min, samples were placed in blocking solution (10% normal goat serum [Sigma Aldrich, St. Louis, MO, USA] in 0.3% PBT) for 30 min. Brains were probed with an anti-TH antibody raised in rabbit (EMD Millipore, Temecula, CA, USA) and diluted 1:100 in blocking solution overnight (12–18 h) at 4°C. The next day, brains were washed fourusing a confocal microscope (Leica Microsyst times in 0.3% PBT for 5 min. Samples were placed in blocking solution for 30 min before incubation with goat anti-rabbit IgG (H + L) secondary antibody conjugated to Alexa Fluor 405 (Life Technologies, Grand Island, NY, USA) diluted 1:200 in blocking solution for a minimum of 2 h at room temperature (RT) to overnight at 4°C. The brains were washed four times in PBS for 5 min, then they were mounted on microscope slides using Vectashield mounting medium (Fisher Scientific, Pittsburgh, PA, USA) for MitoTimer and mitoGFP-only brains or Prolong Diamond mounting medium (Life Technologies, Eugene, OR, USA) for brains containing mCherry-atg8a and mitoGFP. Slides were coded so that the experimenter was blind to genotype and age during image capture and processing.

### Image Capture

Z-stacks of one PPL1 and one PPM3 cell cluster per brain was captured using a confocal microscope (Leica Microsystems, Buffalo Grove, IL, USA) at 630 × with 1.5 zoom (optical section thickness of 1.039 μm and a z-step size of 0.34 μm). A 405 nm laser was used to excite the Alexa-405 secondary antibody conjugate to visualize TH. Both GFP (excitation range, 475–495 nm/emission, 520–560 nm), and the green variant of MitoTimer (peak excitation/emission, 488/518 nm) were excited with a 488 nm laser. A 532 nm laser was used to excite the red variant of MitoTimer, which has an excitation peak of approximately 558 nm and an emission maximum at 583 nm, and mCherry, whose excitation maximum is 587 nm and emission maximum is 610 nm. Each image was captured at a frequency of 400 Hz.

For each fluorophore, gain and offset remained constant when capturing images (e.g., Gain was set at 800 and offset at −1.0 for all green MitoTimer images). In order to account for inconsistencies in immunolabeling and mitoGFP levels in measurements of mitochondrial object size, sphericity and mitochondrial network volume per cell, laser intensity was adjusted, and a pseudo-colored channel indicating overexposure was used so that images could be captured when about 2% of the image was overexposed. For MitoTimer and atg8a protocols, laser intensity was fixed for each fluorophore.

### Digital Image Data Collection

Image Pro Premier 3D (Media Cybernetics Inc., Rockville, MD, USA) was used to measure mitochondrial sphericity, volumes and/or colocalization for all fluorophores in control (*park^+/+^*), *park* heterozygous (*park*^+/25^, referred to as, “*park*^+/−^” in text) and *park* homozygous (*park*^25/25^, referred to as, “*park*^−/−^” in text) flies.

#### Mitochondrial Sphericity and Object Volume Measurements

An automatic intensity threshold for one PPL1 and one PPM3 cluster per brain was obtained for *park^+/+^*, *park*^+/−^ (PPL1 only) and *park*^−/−^ flies expressing the mitoGFP fluorophore construct under the control of TH-GAL4 (*w*^1118^; *UAS-mitoGFP/+*; *TH-GAL4/+* for *park*^+/+^, *w*^1118^; *UAS-mitoGFP/+*; *TH-GAL4, park*^25^/+ for *park*^+/−^, and *w*^1118^; +^*w*1118^; *TH-GAL4*, *park*^25^/UAS-mitoGFP, *park*^25^ for *park*^−/−^), and 3D structures of mitochondrial objects in the GFP channel with fluorescence intensity above threshold were selected. Objects outside of the TH-labeled area were removed so that all objects used for data collection were found within PPL1 DA somas and proximal projections. To determine whether mitochondrial network fragmentation occurs in *park* mutant flies, we measured volume and the inverse of sphericity (six times object volume divided by the object’s diameter surface, or [6V]/DS) for each mitochondrial object. The average mitochondrial object volume and inverse of sphericity for each cluster was recorded for at least 10 clusters per condition. PPL1 mitochondrion size frequency distribution histograms were generated by placing objects into small, medium and large bins based on volume. Bin size was determined by taking the difference of the largest and smallest data point and dividing it by three. Large mitochondrion sphericity analyses include the sphericity data for the largest five percent of all PPL1 mitochondrial objects.

#### MitoTimer Volume Measurements

Three dimensional objects of mitochondrial MitoTimer proteins in the GFP and DSred channels above a standardized intensity threshold were selected from one PPL1 and one PPM3 cluster per brain in *park^+/+^*, *park*^+/−^ and *park*^−/−^ flies expressing MitoTimer under the control of TH-GAL4 (*w*^1118^; +^*w*1118^; *UAS-MitoTimer/TH-GAL4*, for *park*^+/+^; *w*^1118^; +^*w*1118^; *UAS-MitoTimer, park*^25^/*TH-GAL4* for *park*^+/−^; and *w*^1118^; +^*w*1118^; *UAS-MitoTimer, park*^25^/*TH-GAL4, park*^25^ for *park*^−/−^). GFP- and DSred- positive structures outside the TH–labeled region were removed so that all structures used were found within DA somas and proximal projections. Total volume for each channel was obtained by calculating the sum of the volume measurements of individual 3D mitochondrial objects within the TH-labeled regions of one brain cluster. Ratios of total red to total green volume per brain also were calculated (Laker et al., 2014) for at least 11 PPL1 clusters per condition.

#### Autophagosome Formation

In order to determine the relative levels of autophagosome formation, red objects fluorescing above background and between 0.1 nm^3^ and 1 μm^3^ were counted to represent structures of mCherry-atg8a puncta from one PPL1 and one PPM3 (data not shown) region in *park^+/+^*, *park*^+/−^ and *park*^−/−^ flies expressing mCherry-atg8a (atg8a, autophagosome marker) and mitoGFP under the control of TH-GAL4 (*w*^1118^; *UAS-mCherryAtg8a, UAS-mitoGFP/+*; *TH-GAL4/+* for *park*^+/+^, *w*^1118^; *UAS-mCherryAtg8a, UAS-mitoGFP/+*; *TH-GAL4, park*^25^/+ for *park*^+/−^, and *w*^1118^; *UAS-mCherryAtg8a, UAS-mitoGFP/+*; *TH-GAL4 park*^25^/*park*^25^ for *park*^−/−^). We established a standardized method of measuring red objects representing autophagosomes based on qualitative observations of staining patterns. The number of atg8a-positive autophagosomes within TH-labeled somas was divided by the number of TH-positive neurons for at least night brain clusters per condition.

#### PPL1 Dopaminergic Neuron Number

The number of TH-positive neurons in one PPL1 cluster per brain was determined using the Image Pro Premier software manual counting tool to count the number of TH-labeled cells in individual z-stack frames for at least nine PPL1 clusters per condition. The counting tool adds a digital tag that remains on the screen as the observer moves from frame to frame to avoid duplicate counts. TH-positive cells were counted in *park^+/+^*, *park*^+/−^ and *park*^−/−^ flies expressing mCherry-atg8a and mitoGFP under the control of TH-GAL4 (Genotype information is provided above).

#### Autophagosome Recruitment to Mitochondria

We used Image Pro Premier 3D co-localization tool to count the number of mCherry-positive objects (identified as described above) that also were labeled with GFP (mitoGFP) as indicated by a positive Pearson’s correlation coefficient. The number of co-localization events within one TH-labeled PPL1 and one PPM3cluster per brain is reported for at least five clusters for *park^+/+^*, *park*^+/−^ and *park*^−/−^ flies expressing mCherry-atg8a and mitoGFP (genotypes described above).

#### Mitochondrial Network Volume Per Cell

We took the sum of volumes for mitoGFP-positive objects selected for measurements of mitochondrial object volume for each TH-labeled cell within one PPL1 and one PPM3 cluster per brain and divided that value by the corresponding soma volume measurement (indicated by TH-labeling). TH-positive neurons with clearly-defined, TH signal were included the analysis. Data represent at least 15 PPL1 and six PPM3 clusters in *park^+/+^*, *park*^+/−^ and *park*^−/−^ flies expressing mitoGFP under the control of TH-GAL4 (*w*^1118^; *UAS-mitoGFP/+*; *TH-GAL4/+* for *park*^+/+^, *w*^1118^; *UAS-mitoGFP/+*; *TH-GAL4, park*^25^/+ for *park*^+/−^, and *w*^1118^; +^*w*1118^; *TH-GAL4, park*^25^/UAS-mitoGFP, *park*^25^ for *park*^−/−^).

### Western Blotting

Approximately 200–300 *park^+/+^, park^+/−^ and park^−/−^* flies (*w*^1118^, *w*^1118^; *park*^25^/+, and *w*^1118^; *park*^25^/*park*^25^) aged day 1 through 14 PE were transferred to 50 ml conical tubes. Each tube subsequently was frozen in liquid nitrogen and briskly shaken to separate heads from bodies. Frozen heads and bodies were transferred to a no 25 sieve (Fisher Scientific, Waltham, MA, USA) that had been chilled to −80°C. Collected heads were ground thoroughly using a dounce homogenizer (Fisher Scientific, Waltham, MA, USA) containing 200 μl ice-cold fractionation buffer (250 mM Sucrose, 10 mM Tris pH 7.4, 0.15 mM MgCl_2_) freshly supplemented with protease inhibitor cocktail (Roche, Indianapolis, IN, USA). Extracts were centrifuged at 1000× *g* for 5 min at 4°C. Supernatants were centrifuged again at 1000× *g* for 5 min at 4°C. Brain-enriched supernatants were collected, and total protein was determined using the BCA method (Pierce Waltham, MA, USA). Samples were reduced, and 10 μg of protein was separated in 12% Bis-Tris SDS-PAGE gels (Bio-Rad, Hercules, CA, USA) and transferred to a 0.45 μm nitrocellulose membrane (Bio-Rad). The membrane was treated with REVERT™ Total Protein Stain following the recommended protocol (LICOR, Lincoln, NE, USA) and imaged using a 700 nm laser on an Odyssey infra-red imaging system (LICOR). The membrane was then briefly rinsed with Milli-Q^®^ water and blocked under slight agitation in blocking solution (5% non-fat dry milk diluted in Tris-buffered saline with 2% Tween 20 [TBST]) for at least 1 h at RT. In order to identify atg8a, the *Drosophila* gamma amino butyric acid receptor associated protein (GABARAP) ortholog, the membrane was probed with a rabbit monoclonal anti-GABARAP primary antibody (ab109364 lot#GR154272-6; Abcam, Cambridge, MA, USA) diluted 1:10,000 in blocking solution overnight at 4°C under slight agitation. The membrane was washed five times with TBST under moderate agitation at RT and probed with an infrared fluorescent goat-anti-rabbit secondary antibody (IRDye^®^ 800 CW; LICOR, lot#C50602-05) diluted 1:10,000 in blocking solution for at least 1 h under slight agitation at RT. The membrane was washed five times with TBST under moderate agitation at RT followed by one wash in Tris-buffered saline. Washed membranes were allowed to dry in the dark and were subsequently imaged with an 800 nm laser using the Odyssey imaging system.

Membrane images were analyzed using Image Studio Version 4.0 (LICOR). The total protein approach for normalization was used, as evidence suggests commonly used reference proteins can be differentially expressed (Eaton et al., 2013). REVERT™ staining of proteins within the 37–75 kDa range was selected as the total protein signal for each lane (Eaton et al., 2013). A lane normalization factor (LNF) was calculated by dividing the REVERT™ signal for each lane by the REVERT™ signal for the lane that had the highest signal. Atg8a bands at approximately 14 kDa and 12 kDa represent uncleaved and cleaved (activated) forms of atg8a, respectively, and a LNF was calculated for the 14 and 12 kDa bands. Finally, the cleaved or uncleaved LNF for each band was divided by the total protein LNF from the corresponding lane to determine the normalized signal. Total (uncleaved plus cleaved) atg8a and the percent of cleaved atg8a were calculated. Each data point is an average signal of three replicates of the same sample. Samples from four collection days were analyzed.

### Climbing Assays

Climbing (negative geotaxis) assays were performed by placing day 5, 10 or 20 days PE *park^+/+^*, *park*^+/−^ and *park*^+/−^ flies expressing mitoGFP and mCherry-atg8a under the control of TH-GAL4 (genotypes described above) into one of 16 transparent polycarbonate tubes (5 mm diameter, 80 mm length, one fly per tube). A MutiBeam Activity Monitor (TriKinetics Inc. Waltham, MA, USA) held tubes in the vertical position so that 17 independent infrared beams could pass through each tube; the distance between the first and last beam is 51 mm. White yarn was placed just inside of the top and bottom of the polycarbonate tubes in order to trap the fly in the infrared detection zone. Data for five to eight flies per genotype were collected simultaneously, and control flies (*w*^1118^; UAS-mCherryAtg8a, UAS-mitoGFP/+; *TH-GAL4/+*) were included in all climbing trials. Each time a fly crosses an infrared beam, a count is recorded, allowing for determination of the fly’s position in the tube each second for 20 min. “Height climbed” is the distance in mm of a flies’ continuous trajectory from one position to a higher position in the tube. Each time a fly climbs up again after moving downward, a new “height climbed” is recorded. The total height climbed during the 20-min recording period was measured, and divided by number of climbs to calculate average height climbed. A receiver operating characteristic (ROC) curve was generated from mitoGFP, mCherry-atg8a control fly climbing data to distinguish “climbing” from “non-climbing” flies (Microsoft Excel; Microsoft, Redmond, WA, USA). Climbing data for 6–50 flies per condition is reported.

### Statistical Analyses

To determine the effect of the *park* mutation on mitochondrial network morphology and volume, atg8a protein levels, and number of autophagosomes, dopaminergic (DA) neurons and co-localization events, comparisons to control were completed via an ordinary one-way ANOVA followed by Dunnett’s multiple comparisons test when data were normally distributed. For cases in which data did not pass the Shapiro-Wilk normality test, comparisons to control were made using Kruskal-Wallis tests followed by Dunn’s multiple comparisons test. Unpaired *t-tests* were performed to determine the effect of genotype on the sphericity of large mitochondrial objects in PPL1. To determine effects of the *park* mutation and on day Parkinson’s disease (PD) and MitoTimer volumes, comparisons to control (*park*^+/+^ and day 5, respectively) were made via a two-way ANOVA followed by Dunnet’s multiple comparisons test. Fisher’s exact tests were used to determine the effect of the *park* mutation on the frequency distribution of large and small mitochondrial objects. Fisher’s exact tests were also used to determine the distributions of climbing and non-climbing *park* heterozygous and homozygous mutant *Drosophila* compared to that of control, to one another, and within genotypes to measure the effect of time. A Mann-Whitney test comparing ranks was performed to determine the effect of the homozygous *park* mutation on the number of mCherry-atg8a objects colocalized with mitoGFP per cell. For all tests, *α* = 0.05.

## Results

### Vulnerable Dopaminergic Neuron Mitochondria Are Selectively Fragmented and Swollen in Parkin Loss-of-Function Flies

**Figure 1:**
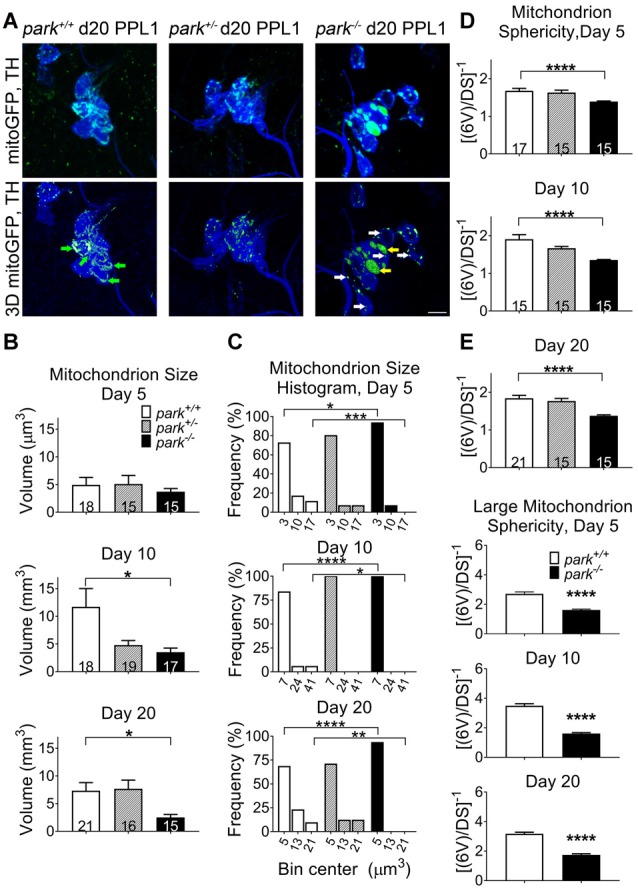
Parkin loss-of-function flies have increased mitochondrial fragmentation and swelling in protocerebral posterior lateral region 1 (PPL1) DA neurons. Brains of *park* mutant flies expressing the mitochondrially targeted green fluorescent protein (mitoGFP) construct in tyrosine hydroxylase (TH)-producing cells were dissected, stained for TH (blue), and fixed on days 5, 10 or 20 post eclosion (PE). Images in **(A)** are representative intensity sums of Z-stacks used as 3D projections for the *park* control (*park*^+/+^), heterozygote (*park*^+/−^) and homozygote (*park*^−/−^) conditions on day 20. Green (mitoGFP) labeling in the bottom row of **(A)** represents 3D isosurfaces that are generated based on signal above an automatic bright threshold. Digital image enhancement steps were standardized for green fluorophores. After using standardized image capture parameters, we used Image Pro Premier 3D image analysis software to measure and categorize the mitochondrial object size **(B)** and inverse of sphericity ([6V]/DS) **(D)** of mitoGFP objects within the TH-labeled regions for one PPL1 region per brain. Mitochondrion size frequency distribution histograms were generated by placing objects into small, medium and large bins based on volume **(C)**. Bin size was determined by taking the difference of the largest and smallest data point and dividing it by three. The angled numbers along the x-axis indicate the bin center, or the value that falls halfway between the smallest and largest values of its bin. We also measured the inverse of sphericity for the largest five percent of objects **(E)**. Green arrows indicate large, interconnected (not swollen) mitochondria. White and yellow arrows indicate fragmented and large, swollen mitochondria, respectively. Numbers in histogram bars indicate the number of PPL1 regions analyzed. For *P < 0.05; for **P < 0.01; for ***P < 0.001; for ****P < 0.0001. Error bars represent standard error of the mean; scale bar represents five microns.

**Figure 2:**
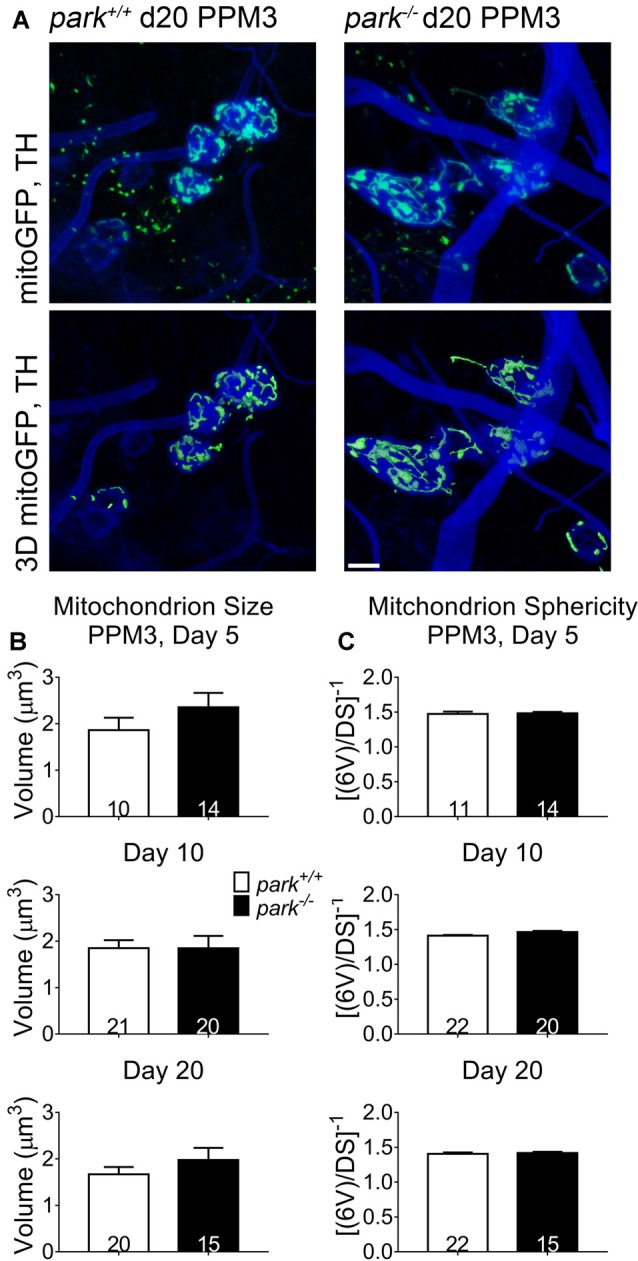
Non-degenerating DA neurons in parkin loss-of-function flies do not have fragmented or swollen mitochondria networks. Brains of *park* mutant flies expressing the mitoGFP construct in TH-producing cells were dissected, stained for TH (blue), and fixed on days 5, 10 or 20 PE. Images in **(A)** are representative intensity sums of Z-stacks used as 3D projections for the *park*^+/+^ and *park*^−/−^ conditions on day 20. Green (mitoGFP) labeling in the bottom row of **(A)** represents 3D isosurfaces that are generated based on signal above an automatic bright threshold. Digital image enhancement steps were standardized for green fluorophores. After using standardized image capture parameters, we used Image Pro Premier 3D image analysis software to measure and categorize the mitochondrial object size **(B)** and inverse of sphericity ([6V]/DS) **(C)** of mitoGFP objects within the TH-labeled regions for one PPM3 region per brain. Numbers in histogram bars indicate the number of regions analyzed. Error bars represent standard error of the mean; scale bar represents five microns.

Since mitochondrial network fragmentation and swelling can be a sign of damage, we measured the size and sphericity of PPL1 and PPM3 DA neuron mitochondria in control flies (*park^+/+^*), *park* heterozygotes (*park*^+/^, PPL1 only) and *park* homozygotes (*park*^−/−^) that also express mitoGFP and TH-GAL4. In contrast to the interconnected, ribbon-shaped mitochondria observed in control (green arrows), most *park*^−/−^ PPL1 mitochondria appeared to be fragmented (Figure 1A, white arrows). A small proportion of large, round mitochondrial objects was observed in the *park*^−/−^ condition (Figure 1A, yellow arrows). These apparently swollen mitochondria were few in number relative to fragmented mitochondria; thus, the average mitochondrial volume per PPL1 region was smaller in the *park*^−/−^ condition on days 10 and 20 (Figures 1A–C). Mitochondria were also more spherical on days 10 and 20, suggesting that *park*^−/−^ PPL1 mitochondrial networks are generally fragmented (Figures 1A,D). The largest five percent of *park*^−/−^ mitochondria were more spherical, indicating that they are swollen, rather than interconnected (Figure 1E). Thus, *park*^−/−^ PPL1 mitochondrial networks are generally fragmented, which can indicate damage and preparation for mitophagy. These mitochondria also show signs of swelling, which not only indicates damage, but also impeded mitophagy. There was no effect of the heterozygous *park* mutation on object sphericity or volume (Figure 1). Interestingly, mitochondrial fragmentation and swelling were not observed in *park*^−/−^ PPM3 neurons, which do not degenerate (Figure 2).

### Mitochondrial Aging Is Advanced in Parkin Mutant PPL1 but Not PPM3 DA Neurons

**Figure 3:**
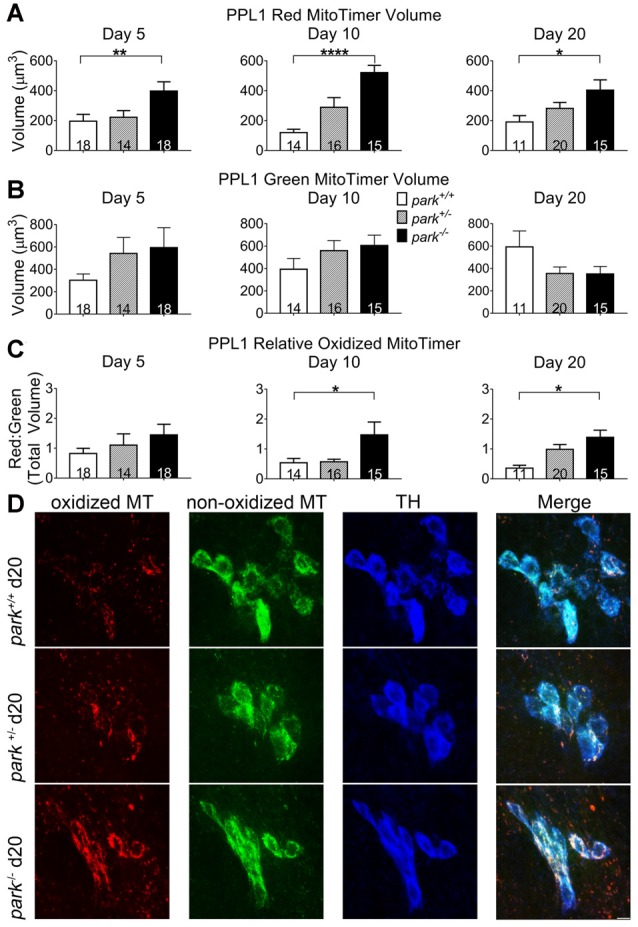
Homozygous parkin loss-of-function flies have increased aged MitoTimer protein levels in PPL1 DA neurons. Brains of *park* mutant flies expressing the MitoTimer age reporter construct in TH-producing cells were dissected, stained for TH (blue), and fixed on days 5, 10 or 20 PE. Using standardized image capture and digital image analysis parameters, we measured the total volume of aged MitoTimer (red; **A**), newly synthesized MitoTimer (green; **B**), and the ratio **(C)** of aged to new MitoTimer within the TH-labeled region for one PPL1 region per brain. Images in **(D)** are digital sums of representative z-stacks for the *park*^−/−^, *park*^+/−^, and *park*^+/+^ conditions on day 20. Digital image enhancement steps were standardized for red and green fluorophores in **(D)**. Numbers in histogram bars indicate the number of PPL1 regions analyzed. For *P < 0.05; for **P < 0.01; for ****P < 0.0001. Error bars represent standard error of the mean; scale bar represents five microns.

**Figure 4:**
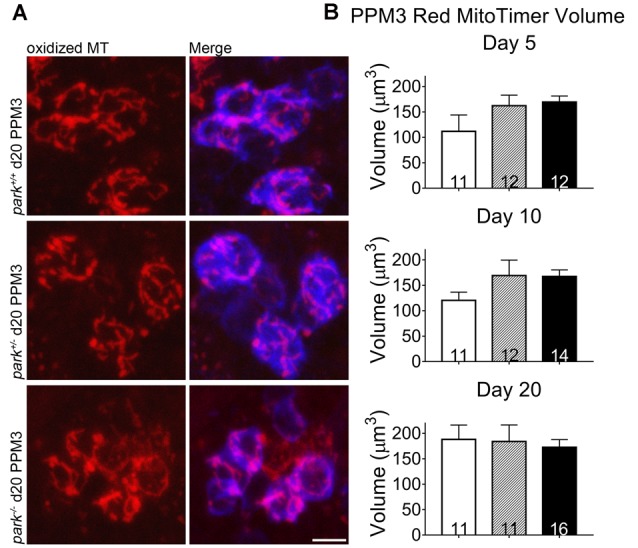
Homozygous parkin loss-of-function non-degenerating DA neurons show signs of normal mitochondrial aging. Brains of *park* mutant flies expressing the MitoTimer age reporter construct in TH-producing cells were dissected, stained for TH (blue), and fixed on days 5, 10 or 20 PE. Using standardized image capture and digital image analysis parameters, we measured the total volume of aged MitoTimer (red) within the TH-labeled region for one PPM3 region per brain **(A,B)**. Images in **(A)** are digital sums of representative z-stacks for the *park*^−/−^, *park*^+/−^, and *park*^+/+^ conditions on day 20. Digital image enhancement steps were standardized for red fluorophores. Numbers in histogram bars indicate the number of PPM3 regions analyzed. Error bars represent standard error of the mean; scale bar represents five microns.

Mitochondrial damage and network fragmentation can be caused by uncontrolled levels of macromolecule oxidation. We found that *park^−/−^* flies expressing MitoTimer and TH-GAL4 had greater volumes of red, aged MitoTimer per PPL1 cluster than *park*^+/+^ flies on day 5, 10 and day 20 PE (Figures 3A,D). There were no changes in levels of green (newly synthesized) MitoTimer, and the ratio of red to green total volume per cluster was increased on days 10 and 20 (Figures 3B–D). There was no effect of day PE on MitoTimer measurements, indicating that as flies age, mitochondrial turnover is consistent. Levels of red MitoTimer in PPM3 were unaffected by the *park* mutation, suggesting that only susceptible DA neurons show signs of aberrant mitochondrial aging (Figure 4). Thus, accelerated mitochondrial aging, likely caused by increased mitochondrial protein oxidation, may contribute to the *park* mutant PPL1 degeneration.

### Autophagosome-Promoting atg8a Activation and Autophagosome Numbers Are Not Affected by the *park* Mutation

**Figure 5:**
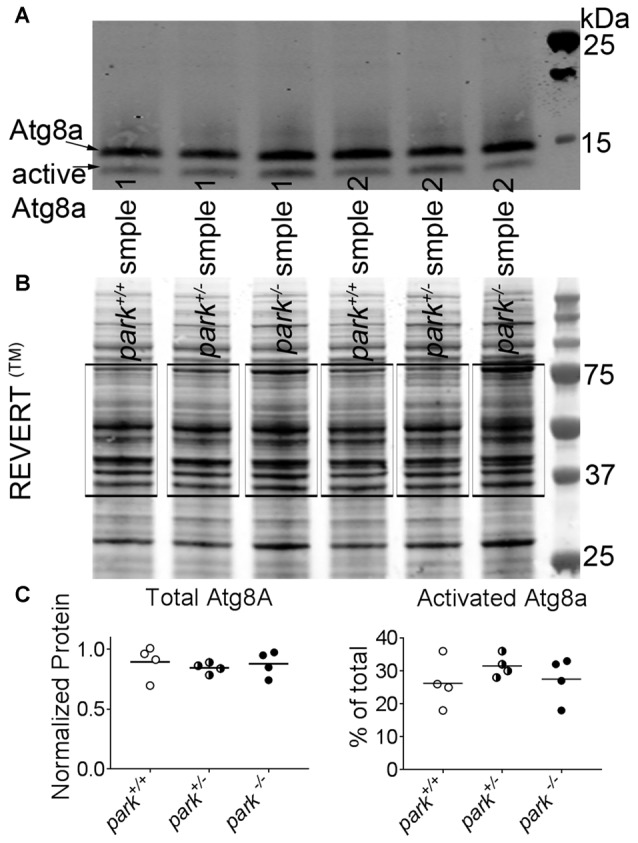
Levels of active and inactive autophagy 8a (atg8a) are unchanged in *park* mutant fly heads. Ten microgram of homogenized *park*^−/−^, *park*^+/−^, or *park*^+/+^ fly brain fractions per lane was transferred to a nitrocellulose membrane, which was incubated in REVERT^™^ protein stain and imaged using an automatic bright threshold. The REVERT™ stain was washed, and the membrane was probed with a rabbit anti-gamma amino butyric acid receptor associated protein (GABARAP) primary antibody (recognizing *Drosophila* atg8a) followed by an anti-rabbit secondary antibody with an infrared fluorescent conjugate (IRDye^®^ 800CW, LICOR) **(A)**. Total protein was determined by measuring REVERT™ fluorescence between 75 kDa and 37 k Da for each lane (**B**, black boxes). Fluorescence signal for each lane was divided by the signal for the lane that had the highest intensity. For atg8a measurements, the signals from the inactive 14 kDa and the active 12 kDa bands were divided by the corresponding signal for the band that had the highest intensity. To control for loading, these values were divided by the relative REVERT™ total protein value for each lane (Eaton et al., 2013). There was no effect of the *park* mutation on total or activated atg8a levels **(C)**. Each data point is an average of three technical replicates for one collection day; lysates were generated on four different collection days (indicated in histogram bars).

**Figure 6:**
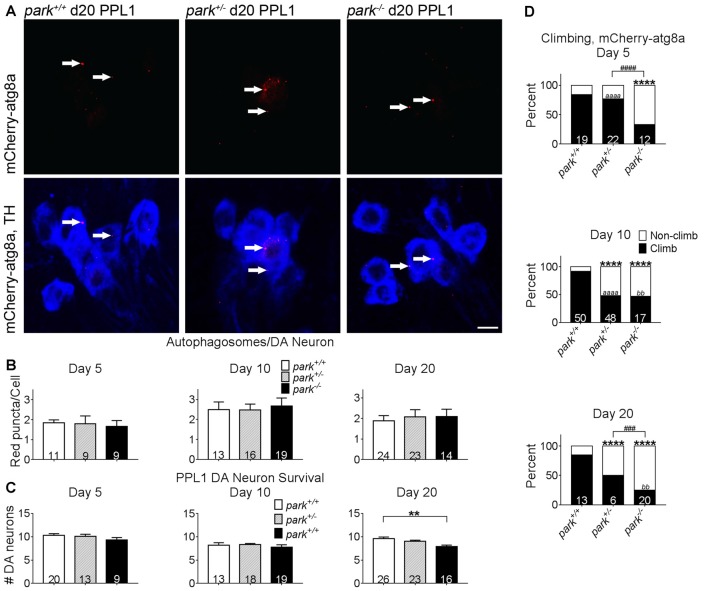
Exogenous expression of atg8a in TH-producing neurons does not ameliorate DA degeneration or climbing deficits in *park* mutant flies. Brains of flies expressing the mitoGFP and mCherry-atg8a constructs in TH-producing cells were dissected, stained for TH (blue), and fixed on days 5, 10 or 20 PE. Images in **(A)** are digital sums of representative z-stacks for day 20 *park*^+/+^, *park*^+/−^, *park*^−/−^ PPL1; digital image enhancement steps were standardized for the mCherry (red) fluorophore. Using standardized image capture and digital image analysis parameters, we identified and counted the number of atg8a puncta (white arrows) within TH-labeled regions and divided that value by the number of TH-positive cells for one PPL1 region per brain **(B)**. We also counted the number of TH-positive cells per cluster **(C)**. **(D)** Climbing assays were performed by placing individual flies expressing mitoGFP and mCherry-atg8a into polycarbonate tubes; the fly’s position in the tube was recorded each second for 20 min. The total distance climbed during the 20-min recording period was measured, and divided by number of climbs to calculate the average height climbed. A receiver operating characteristic (ROC) curve was generated from mitoGFP, mCherry-atg8a control fly climbing data to distinguish “climbing” from “non-climbing” flies. Numbers in histogram bars indicate the number of PPL1 regions analyzed for **(B,C)**; for **(D)**, number in histogram bars represent the number of flies tested. For ** and ^bb^*P* < 0.01; for ^###^*P* < 0.001; ^####^*P* < 0.0001; for **** and ^aaaa^*P* < 0.0001. Error bars represent standard error of the mean; scale bar represents five microns.

To facilitate autophagosome formation, atg8a is cleaved at the C-terminal before it is conjugated to phosphatidylethanolamine of pre-autophagic vesicles (Kirisako et al., 2000). In cell lines that express little or no parkin, autophagosome formation is unaffected by exogenous parkin (Ding et al., 2010). In order to address whether disruptions in autophagosome formation occur in an *in vivo* parkin loss-of-function model, brain-enriched fractions from day 1 to 14 PE *park*^+/+^, *park*^+/−^ and *park*^−/−^ flies were used for western blotting. We observed no effect of genotype on total atg8a or percent activated relative to total (Figure 5). In order to address whether disruptions in autophagosome formation may be limited to susceptible neurons, we counted autophagosomes in *park* mutant flies that express mCherry-atg8a and TH-GAL4. Similar numbers of mCherry-atg8a positive autophagosomes per cell (PPL1) or per cluster (PPM3; data not shown) were detected in all three genotypes (Figures 6A,B). Thus, parkin does not appear to play a role in *Drosophila* brain atg8a activation, or PPL1/PPM3 DA neuron autophagosome formation.

### Promotion of Autophagy Initiation Fails to Prevent PPL1 Dopaminergic Neurodegeneration and Climbing Behavior Deficits in *park* Mutants

Studies have shown that exogenous expression of autophagosome-forming atg8a can prevent DA neuron mitochondrial membrane depolarization and degeneration in flies aged to at least day 15 PE (Burman et al., 2012). TH-driven mCherry-atg8a expression does not prevent DA neuron degeneration in *park^−/−^* flies (Figure 6C). Negative geotaxis is a motivated behavior that is facilitated in part by DA neurons (Riemensperger et al., 2011; Barone and Bohmann, 2013). Deficiencies in climbing behavior have been detected in *park* homozygous and heterozygous mutants (Greene et al., 2003; Chambers et al., 2013), and these deficiencies persisted in *park*^+/−^ flies on days 10 and 20, and in *park*^−/−^ flies on days 5, 10 and 20 (Figure 6D). Homozygous *park* mutants had decreased climbing proficiency compared to *park* heterozygotes on days 5 and 20. Climbing deficits worsened from day 5 to day 10 in *park*^+/−^ and from day 10 to 20 in *park*^−/−^ (Figure 6D). Thus, the exogenous expression of mCherry-atg8a in vulnerable DA neurons fails to rescue to the *park* mutant phenotype.

### Mitophagy Initiation Is Selectively and Transiently Decreased in Homozygous *park* Mutant PPL1 DA Neurons

**Figure 7:**
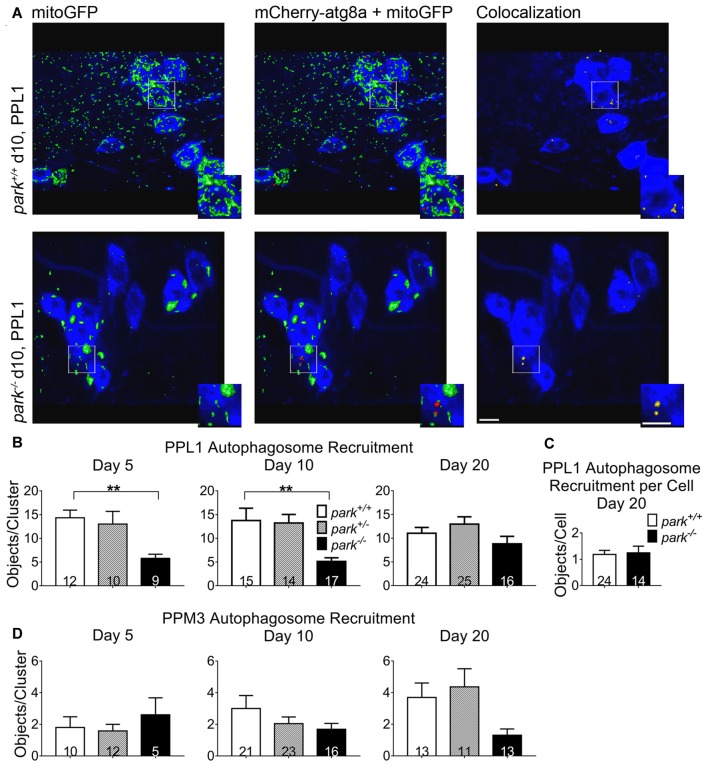
Autophagosome recruitment to mitochondria is selectively and transiently reduced in *park* mutant PPL1. Brains of *park* mutant flies expressing the mitoGFP and mCherry-atg8a constructs in TH-producing cells were dissected, stained for TH (blue), and fixed on days 5, 10 or 20 PE. Red and green objects in **(A)** represent 3D isosurfaces that are generated based on signal above background for representative z-stacks of the *park*^−/−^ and *park*^+/+^ conditions on day 10. TH signal is represented as a summary of the z-stack. Insets highlight mitochondria that are colocalized with atg8a **(A)**. Using standardized image capture and digital image processing analysis, we identified and counted the number of atg8a puncta that colocalized with mitoGFP according to a positive Pearson’s correlation coefficient within the TH-labeled regions for one PPL1 and one PPM3 region per brain (**B,D**, respectively). Because there was a decrease in PPL1 neurons in *park*^−/−^ flies and no change in colocalizations per cell on day 20, we divided the number of colocalizations by the number of TH-positive (DA) neurons for the corresponding PPL1 cluster in day 20 *park*^+/+^ and *park*^−/−^ **(C)**. Numbers in histogram bars indicate the number of DA regions analyzed. For **P < 0.01. Error bars represent standard error of the mean; scale bars represent five microns.

As an initiating step toward mitophagy, autophagosomes are recruited to damaged mitochondria. Whether parkin is required for autophagosome recruitment seems to depend on the experimental model and method (Narendra et al., 2008; Ding et al., 2010; Allen et al., 2013; Strappazzon et al., 2015). In *park* mutant flies expressing mCherry-atg8a, mitoGFP and TH-GAL4, we found that the number of mCherry puncta colocalizing with GFP was decreased in *park*^−/−^ PPL1 DA clusters on days 5 and 10 PE (Figures 7A,B). Interestingly, autophagosome recruitment in *park*^−/−^ was similar to that of *park*^+/+^ on day 20, suggesting that compensatory mechanisms for parkin-independent autophagosome recruitment are triggered after day 10, as the phenotype worsens. Fewer DA neurons were detected on day 20, so we measured the number of colocalized objects per cell and again found no difference (Figure 7C). Harboring one functional copy of the *park* gene was sufficient for maintaining control levels of autophagosome recruitment, and recruitment was unaffected in PPM3 (Figure 7D). Thus, parkin appears to play a role in, but is not required for, mitophagy initiation in vulnerable PPL1 neurons. It does not seem to be involved in autophagosome recruitment for non-degenerating DA neurons. These data suggest that decreased autophagosome recruitment may be a primary contributor to *park* mutant PPL1 degeneration.

### Decreased Mitophagy Initiation in PPL1 DA Neurons Does Not Cause an Increase in Mitochondrial Network Volume

**Figure 8:**
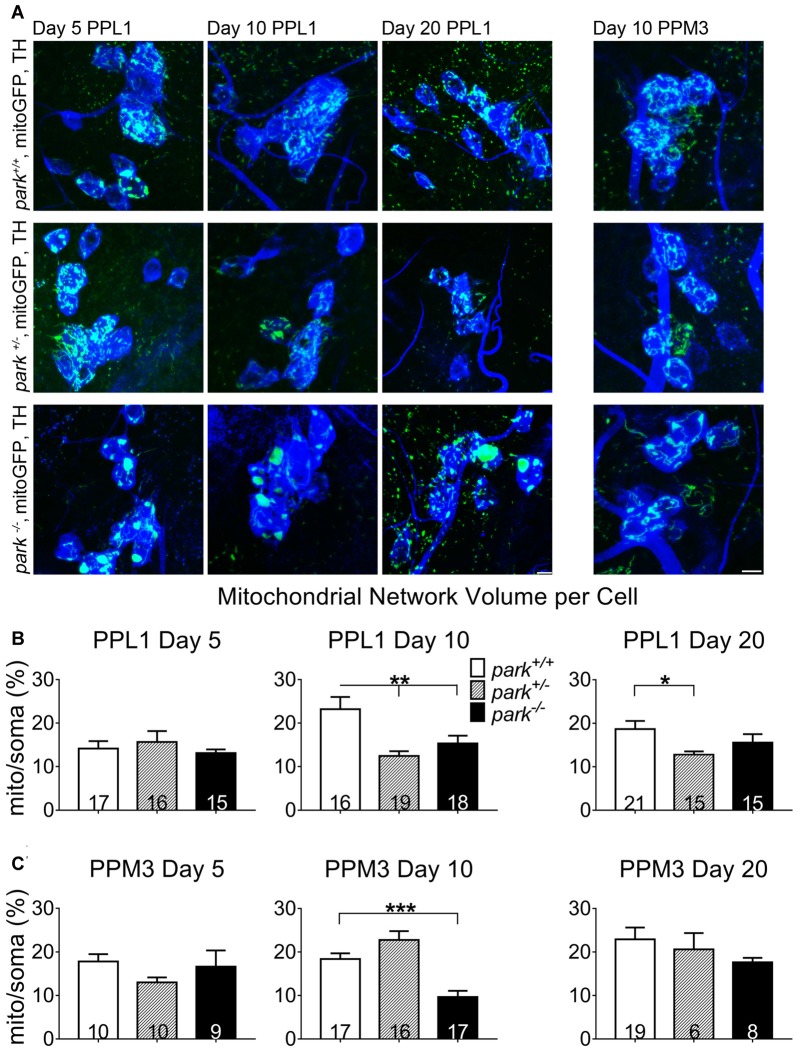
Mitochondrial network volume is decreased in *park* mutant fly DA cells. Brains of *park* mutant flies expressing only the mitoGFP construct in TH-producing cells were dissected, stained for TH (blue), and fixed on days 5, 10 or 20 PE. Using standardized image capture and digital image analysis, we took the sum of the volume of all mitoGFP objects fluorescing above an automatically generated brightness threshold that were within the TH-labeled region for one cell, and divided that value by the volume of the corresponding TH-labeled soma and proximal projections for one region per brain **(B,C)**. Images in **(A)** are sums of representative z-stacks for *park*^+/+^, *park*^+/−^ and *park*^−/−^ day 5, 10 and 20 PPL1 and day 10 PPM3. Digital image enhancement steps were standardized for green fluorophores. Numbers in histogram bars indicate the number of regions analyzed. For *P < 0.05; for **P < 0.01; for ***P < 0.001. Error bars represent standard error of the mean; scale bar represents five microns.

Because autophagosome recruitment (mitophagy induction) is decreased in *park* homozygotes, we hypothesized that mitochondrial network volume would be increased in *park*^−/−^ flies. However, we found that mitochondrial network volume per cell was similar in mitoGFP and TH-GAL4-expressing *park*^+/+^ and *park*^−/−^ on days 5 and 20. Intriguingly, PPL1 mitochondrial network volume was decreased in *park*^−/−^ on day 10 (Figures 8A,B), when autophagosome recruitment was decreased, suggesting that alternative forms of mitochondrial turnover are initiated. PPL1 mitochondrial network volume was decreased in *park*^+/−^ on days 10 and 20 (Figure 8B). Thus, signs of mitochondrial damage are evident in heterozygote PPL1 but not PPM3 neurons. Mitochondrial network volume was decreased in *park*^−/−^ PPM3 on day 10, and it recovers on day 20 (Figure 8C).

## Discussion

We have shown that mitochondria in vulnerable DA neurons selectively harbor signs of damage in an *in vivo* parkin loss-of-function model. Our experiments were performed on days 5, 10 and 20 PE in order to determine whether signs of mitochondrial damage and disruptions in mitophagy are associated with *park* mutant phenotypes (Greene et al., 2003; Chambers et al., 2013). *Park*^−/−^ PPL1 but not PPM3 DA neurons have fragmented mitochondrial networks, swollen mitochondria and accelerated mitochondrial aging, which could result from mitochondrial protein oxidation. Inability to maintain homeostatic mitochondrial respiration and/or ROS levels could result from decreased proteasome- or lysosome- mediated turnover of mitochondrial proteins (Lim et al., 2005; Whitworth et al., 2005; Mortiboys et al., 2008; Burman et al., 2012; Vincow et al., 2013). The *park* mutation does not affect atg8a activation in the fly brain or autophagosome number in DA neurons, and exogenous expression of mCherry-atg8a in DA neurons does not prevent PPL1 degeneration or motivated behavior. Mitophagy initiation is unaffected in non-degenerating DA neurons, but it is transiently decreased in *park*^−/−^ PPL1. Our data suggest that parkin’s role in autophagosome recruitment differs among DA regions, and that decreased mitochondrial recruitment and accelerated aging may be principle causes of neurodegeneration in the absence of parkin. Recovery of autophagosome recruitment implies that alternative mechanisms for mitophagy initiation are triggered as mitochondrial pathology and deficits in motivated behavior persist. Mitochondrial network volumes are decreased in *park* mutants, even when mitophagy initiation is decreased, suggesting that alternative forms of mitochondrial turnover may be involved. PPL1 neurons may be selectively vulnerable because they harbor damaged mitochondria in a system where mitophagy initiation is impaired.

Parkin can promote turnover of protein substrates by directing them to lysosomes via Lysine 63 ubiquitination (Doss-Pepe et al., 2005; Lim et al., 2005; Olzmann et al., 2007; Gegg et al., 2010; Poole et al., 2010; Ziviani et al., 2010; Chan et al., 2011; Glauser et al., 2011; Rakovic et al., 2011). Our data show for the first time that parkin plays a non-essential role in autophagosome recruitment to mitochondria in susceptible DA neurons *in vivo*, and that parkin is not required for autophagosome recruitment in non-degenerating DA neurons. Interestingly, mitochondrial network volume is decreased when autophagosome recruitment is decreased or unaffected, and levels of green, newly synthesized MitoTimer are steady. This suggests that an autophagosome-independent method of mitochondrial turnover may be at work (Vincow et al., 2013). Future experiments could determine whether PINK1 levels or activity are augmented to recover PPL1 autophagosome recruitment in the absence of parkin (Lazarou et al., 2015). Since homozygote PPL1 neurons degenerate by day 20, it may be that surviving DA neurons are more robust, and/or have more efficient mitochondrial turnover. Nonetheless, PPL1 neurons that are present on day 20 show signs of mitochondrial damage. Systemic exogenous expression of atg8a previously has been shown to restore mitochondrial membrane potential, prevent PPL1 DA neuron loss, improve lifespan, and promote resistance to oxidative stress and the accumulation of ubiquitinated proteins in *Drosophila* (Simonsen et al., 2008; Burman et al., 2012). We detected no change in atg8a activation, and a small, perhaps delayed decrease in PPL1 DA neuron number in *park* homozygotes compared to control flies expressing TH-driven mCherry-atg8a. The question remains whether exogenous atg8a expression could reduce mitochondrial fragmentation or relative red MitoTimer ratios in *park* PPL1. We observed no change in mitophagy initiation in *park*^−/−^ PPM3 or in *park*^+/−^ PPL1, neither of which degenerate. Yet *park*^+/−^ PPL1 mitochondria show signs of dysfunction at time points when motor deficits occur (Chambers et al., 2013). Thus, degeneration of susceptible *Drosophila* dopaminergic neurons may result from a combination of decreased parkin-mediated mitophagy and premature mitochondrial aging induced by oxidative stress. In *Drosophila*, parkin has been shown to facilitate autophagosome-independent respiratory chain enzyme subunit turnover; however, whether these proteins are targeted to the proteasome or delivered to the lysosome in mitochondria-derived vesicles is unclear (Soubannier et al., 2012; Vincow et al., 2013). Parkin facilitates proteasome-mediated turnover of inner membrane morphology and respiratory chain enzyme function stabilizer myeloid cell leukemia 1 (MCL-1), pro-apoptotic factor, Bax, and translocase of the outer membrane (TOM) subunits (Chan et al., 2011; Yoshii et al., 2011; Johnson et al., 2012; Perciavalle et al., 2012; Charan et al., 2014). The TOM complex must function at optimal capacity in order to maintain mitochondrial functions, since all but 13 mitochondrially-expressed proteins must be imported. Whether disruption in TOM subunit turnover contributes to age acceleration and/or fragmentation of mitochondria as observed here remains to be elucidated. Parkin can promote mitochondrial network connectivity, perhaps by facilitating turnover of mitochondrial fission protein drp1 and its outer membrane receptor, fis1 (Lutz et al., 2009; Wang H. et al., 2011). On the other hand, exogenous parkin expression can promote mitochondrial fragmentation (Ziviani et al., 2010; Buhlman et al., 2014), and the *Drosophila* parkin mutant phenotype improves with over-expression of drp1 or decreased expression of fusion proteins mitofusin 2 (mfn2) or opa1 (Deng et al., 2008; Poole et al., 2008). Parkin-overexpression may induce mitochondrial network fragmentation via K48 ubiquitination of outer membrane fusion proteins mfn1 and mf2 and/or microtubule associated motor protein, miro-1, a Rho GTPase that connects mitochondria to microtubules and facilitates their motility (Gegg et al., 2010; Poole et al., 2010; Ziviani et al., 2010; Chan et al., 2011; Glauser et al., 2011; Rakovic et al., 2011; Wang X. et al., 2011; Birsa et al., 2014; Kazlauskaite et al., 2014). Mitochondrial fusion can buffer the effects of minor damage to mitochondria (Rossignol et al., 2004; Tondera et al., 2009; Rambold et al., 2011), and fragmentation facilitates mitophagy when mitochondrial networks are damaged beyond repair. Cell-specific energy requirements may determine whether mitochondrial networks become interconnected or fragmented in the absence of parkin (Rafelski, 2013); they may also determine the demand for mitophagy.

## Conclusion

The role of parkin in mitophagy and mitochondrial homeostasis in various cell types has been extensively described prior to this study. We have provided evidence that mitochondrial network fragmentation, age acceleration, and decreased mitophagy initiation may be directly implicated in DA neurodegeneration *in vivo*. Changes in mitophagy initiation are not associated with non-degenerating DA neurons. Thus, our data suggest that *Drosophila* PPL1 neurons are selectively dependent on parkin-mediated mitophagy. These vulnerable neurons appear to invoke compensation for the loss of parkin by triggering parkin-independent mitophagy and alternative forms of mitochondrial turnover. Critical differences in gene expression, energy demands and synaptic partners of PPL1 and PPM3 remain to be explored. It is of note that MitoTimer, mitoGFP and mCherry-atg8a object measurements were limited to those distributed in cell bodies and proximal projections. Mitochondrial morphology, respiration and turnover may differ among regions like soma, axons, axon terminals and dendrites (Chang et al., 2006; Ashrafi et al., 2014). Nonetheless, our results provide new insight on the effect of the *park* mutation on susceptible DA neuron mitochondria *in vivo*, and promote a better understanding of the mechanisms of degeneration caused by parkin loss-of-function.

## Author Contributions

JC was involved in protocol development for measurements of mitochondrial shape and volume, and she performed and analyzed data for Figures 1, 6. She also contributed to the writing of the manuscript. SG-L was involved in protocol development for MitoTimer and mCherry-Atg8 analyses; and performed and analyzed data for Figures 2, 4, 5, and contributed to writing the manuscript. GBC generated genetic crosses for *Drosophila* strains, provided critical consultation on project development and critically edited the manuscript. AJ performed western blotting experiments and analyzed western blotting data and also contributed to writing the manuscript. SO performed and analyzed climbing data for day 5 *Drosophila*. CHJ performed PCR to confirm park25 genotypes. LMB supervised the project, generated the figures and wrote the manuscript.

## Conflict of Interest Statement

The authors declare that the research was conducted in the absence of any commercial or financial relationships that could be construed as a potential conflict of interest.

## Abbreviations

atg8a: autophagy 8a
DA: dopaminergic
GABARAP: gamma amino butyric acid receptor associated protein
LC3: light chain 3
LNF: lane normalization factor
mfn2: mitofusin 2
mitoGFP: mitochondrially-targeted green fluorescent protein
*park+/+*: control *park Drosophila*
park+/−: heterozygous *park* loss-of-function *Drosophila*
*park*−/−: homozygous *park* loss-of-function *Drosophila*
PBS: phosphate-buffered saline
PBT: PBS containing 0.3 percent Triton X-100
PD: Parkinson’s disease
PE: post eclosion
PPL1: protocerebral posterior lateral region 1
ROC: receiver operating characteristic
TH: tyrosine hydroxylase
TOM: outer mitochondrial membrane translocase
UAS: upstream activating sequence
VDAC: voltage-dependent anion channel.
